# Histone modifications and metabolic reprogramming in tumor-associated macrophages: a potential target of tumor immunotherapy

**DOI:** 10.3389/fimmu.2025.1521550

**Published:** 2025-05-01

**Authors:** Yiting Xu, Han Zhang, Dengyun Nie

**Affiliations:** ^1^ The Second Clinical Medical College, Nanjing Medical University, Nanjing, China; ^2^ School of Chinese Medicine, Nanjing University of Chinese Medicine, Nanjing, Jiangsu, China; ^3^ Nanjing Hospital of Chinese Medicine Affiliated to Nanjing University of Chinese Medicine, Nanjing, Jiangsu, China

**Keywords:** histone modifications, tumor-associated macrophages, tumor microenvironment, immunotherapy, epigenetics

## Abstract

Histone modifications, including methylation, acetylation, lactylation, phosphorylation, ubiquitination, SUMOylation, ADP-ribosylation, and crotonylation, critically regulate tumor-associated macrophages (TAMs) polarization by modulating gene expression and functional states. Reprogramming TAMs from M2 to M1 phenotypes through epigenetic targeting has emerged as a promising strategy to enhance anti-tumor immunity and improve the efficacy of cancer immunotherapy. This review explores the role of histone modifications in TAM biology, their interplay with metabolic reprogramming, and the opportunities and challenges in developing epigenetic-based therapies to advance cancer immunotherapy.

## 1 Introduction

Tumor-associated macrophages (TAMs) being a key component of the tumor microenvironment (TME), which are increasingly recognized for their critical role in shaping tumor immunity and influencing therapeutic outcomes. TAMs exhibit remarkable plasticity, polarizing into either pro-inflammatory M1 or immunosuppressive M2 phenotypes, which respectively inhibit or promote tumor progression. Recent advances have highlighted the importance of histone modifications, especially various modifying enzymes, in regulating TAM polarization and function. These epigenetic mechanisms offer promising targets for reprogramming TAMs from immunosuppressive M2 phenotypes to anti-tumor M1 states, thereby enhancing the efficacy of cancer immunotherapy. However, challenges such as tumor heterogeneity, resistance mechanisms, and the complex interplay between histone modifications and metabolic pathways remain significant hurdles. By summarizing recent findings, this review is structured to first examine the mechanisms of histone modifications in TAM polarization, followed by their metabolic regulation, and finally their therapeutic implications and challenges. This review aims to provide a comprehensive overview of the role of histone modifications in TAM biology, explore their interaction with metabolic reprogramming, and discuss the potential of epigenetic-based strategies to reshape the TME.

## 2 Macrophages and tumor-associated macrophages

Macrophages are well known as versatile and essential components of the immune system, playing crucial roles in both innate and adaptive immunity. TAMs are a heterogeneous population of immune cells that originate from circulating monocytes and infiltrate the TME (1). TAMs play a pivotal role in modulating tumor progression and can exhibit diverse functions depending on the cytokine milieu and signals received from the tumor. Macrophages are generally classified into two main phenotypes: classically activated M1-type macrophages and alternatively activated M2-type macrophages. The differentiation of macrophages into M1 or M2 is referred to as polarization.

### 2.1 M1-type macrophages

M1-type macrophages respond to danger signals transmitted by bacterial products or interferon-γ (IFN-γ), which attract and activate cells of the adaptive immune system. Additionally, M1-type macrophages are typically activated by lipopolysaccharide (LPS), granulocyte-macrophage colony-stimulating factor (GM-CSF), and tumor necrosis factor α (TNF-α). Several pathways promote macrophage polarization to the M1 phenotype, including the IRF/STAT, LPS/TLR4, and NF-κB/PI3K pathways. M1-type macrophages are characterized by strong antigen-presenting activity and the secretion of proinflammatory cytokines such as interleukin-1β (IL-1β), IL-6, IL-12, IL-18, IL-23, and TNF-α. Furthermore, M1-type macrophages also secrete chemokines such as CXCL-9 and CXCL-12. Moreover, M1-type macrophages express high levels of major histocompatibility complex II (MHC II), CD68, CD80, and CD86 and so on (2).

M1-type macrophages exert anti-tumor effects by distinguishing tumor cells from normal cells and ultimately eliminating tumor cells. Research indicates that M1-type macrophages utilize two distinct mechanisms to eliminate tumor cells. First, M1-type macrophages directly mediate cytotoxicity against tumor cells by releasing tumor-killing molecules such as reactive oxygen species (ROS) and nitric oxide (NO) (3). This process is relatively slow, typically taking 1 to 3 days. The second mechanism involves antibody-dependent cell-mediated cytotoxicity, which allows for a faster response, generally killing tumor cells within a few hours (4). Antibody-dependent cell-mediated cytotoxicity requires the participation of anti-tumor antibodies.

### 2.2 M2-type macrophages

M2-type macrophages can be activated by parasites, fungal infections, immune complexes, apoptotic cells, and cytokines such as IL-4, IL-13, IL-25, IL-33, and TGF-β. In contrast to the classically activated isoform, the alternatively activated isoform down-regulates IL-1, IL-6, IL-12, IL-23, and TNF-α while up-regulating IL-10. M2-type macrophages express chemokines such as CCL1, CCL17, CCL18, CCL22, and CCL24. The M2 phenotype is characterized by the expression of CD163, CD204, CD206, and CD209. Additionally, M2-type macrophages express numerous scavenger receptors, which are associated with the high-level expression of IL-10, IL-1β, vascular endothelial growth factor (VEGF), and matrix metalloproteinases (MMPs) (2).

**Figure 1:**
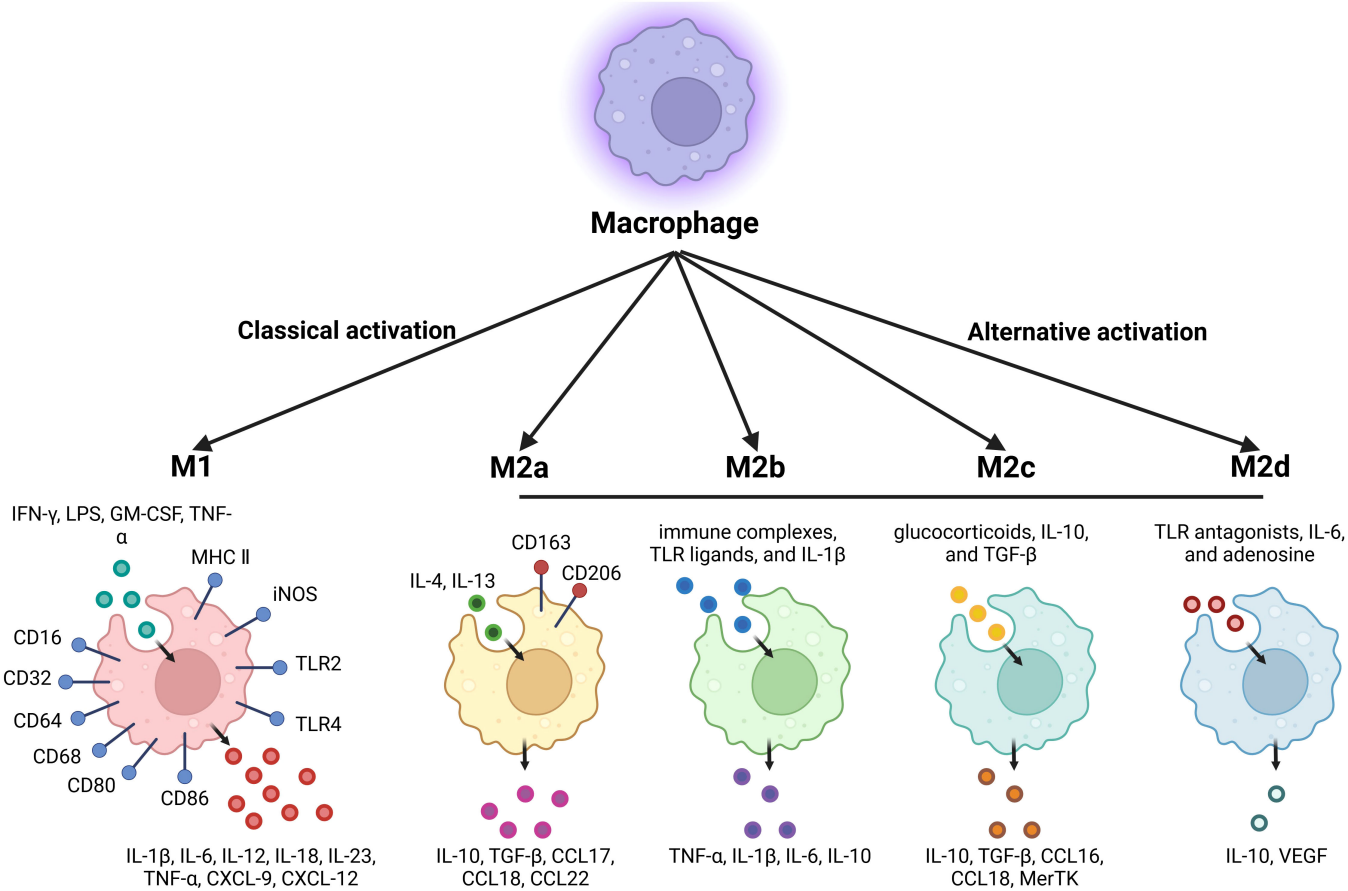
Summary of classically activated M1-type macrophages and alternatively activated M2-type macrophages.

Four M2 subtypes are known to respond to different stimuli: M2a, M2b, M2c, and M2d (5). These subtypes differ in their cell surface markers, secreted cytokines, and biological functions (6). M2a macrophages are activated by IL-4 or IL-13. IL-4 promotes the expression of CD206 and further up-regulates IL-10, TGF-β, CCL17, CCL18, and CCL22, which are known to promote cell growth, tissue repair, and endocytosis (7). M2b macrophages are activated by immune complexes, TLR ligands, and IL-1β. Upon activation, M2b macrophages release both pro-inflammatory and anti-inflammatory cytokines, including TNF-α, IL-1β, IL-6, and IL-10. M2b macrophages play a critical role in regulating immune responses and inflammation (8). M2c macrophages can be activated by glucocorticoids, IL-10, and TGF-β, and are characterized by high expression of the anti-inflammatory cytokine IL-10, the pro-fibrotic factor TGF-β, CCL16, CCL18, and Mer receptor tyrosine kinase (MerTK), which promotes the endocytosis of apoptotic cells (9). M2d macrophages are activated by TLR antagonists, IL-6, and adenosine, with adenosine promoting the expression of IL-10 and VEGF, thereby exacerbating angiogenesis and tumor progression (10) (**Figure 1**; **Table 1**). Collectively, M2-type macrophages function to remove debris, promote angiogenesis, facilitate tissue reconstruction and injury repair, and promote tumorigenesis and progression.

**Table 1:** Macrophage activators and biomarkers.

Types	Activators	Biomarker	
		Secretion	Expression
M1	IFN-γ, LPS, GM-CSF, TNF-α	IL-1β, IL-6, IL-12, IL-18, IL-23, TNF-α, CXCL-9, CXCL-12	CD16, CD32, CD64, CD68, CD80, CD86, MHC II, iNOS, TLR2, TLR4
M2a	IL-4, IL-13	IL-10, TGF-β, CCL17, CCL18, CCL22	CD163, CD206
M2b	immune complexes, TLR ligands, and IL-1β	TNF-α, IL-1β, IL-6, IL-10	
M2c	glucocorticoids, IL-10, and TGF-β	IL-10, TGF-β, CCL16, CCL18, MerTK	
M2d	TLR antagonists, IL-6, and adenosine	IL-10, VEGF	

### 2.3 The effectors of tumor-associated macrophages polarization in the tumor microenvironment

M1-type TAMs are generally regarded as tumor-killing macrophages that primarily promote anti-tumor immunity. In contrast, M2-type TAMs are immunosuppressive and facilitate tissue repair and tumor progression. Both M1 and M2 TAMs are present at all stages of tumor progression (11). M1-type TAMs predominate in the early stages, while M2-type TAMs are more prevalent in the middle and late stages. As the tumor progresses, M1-type TAMs gradually polarize into M2-type TAMs, and an increase in the number of M2-type TAMs indicates a poor prognosis. The balance between M1 and M2 TAMs is critical for determining the overall immune response within the TME (12).

M2-type TAMs with the high-level expression of MMPs, such as MMP-2 and MMP-9, degrade the extracellular matrix, facilitating angiogenesis and tumor cell invasion. Similarly, M2d TAMs secrete VEGF, which promotes the formation of new blood vessels, supplying tumors with oxygen and nutrients. VEGF also enhances vascular permeability, facilitating tumor cell invasion and metastasis. Additionally, M2-type TAMs exert immunosuppressive effects in the tumor microenvironment by secreting a series of cytokines and chemokines, as described below: IL-10 is a key immunosuppressive cytokine, which inhibits the activation of dendritic cells and T cells, suppresses the production of pro-inflammatory cytokines, and promotes the differentiation of regulatory T cells (Tregs), leading to immune evasion. TGF-β suppresses the anti-tumor activity of cytotoxic T cells and natural killer (NK) cells, while promoting the differentiation of Tregs. It also contributes to the epithelial-mesenchymal transition, facilitating tumor cell invasion and metastasis. These chemokines (CCL16, CCL17, CCL18, and CCL22) recruit immune-suppressive cells, such as Tregs and myeloid-derived suppressor cells (MDSCs), to the TME, further enhancing immune evasion.

Although M2-type TAMs dominate in most advanced cancers, M1-type TAMs can also be present, especially in the early stages of tumor development. M1-type TAMs secrete factors that exert anti-tumor effects: IL-12, TNF-α, and IL-1β activate cytotoxic T cells and NK cells, and CXCL9 recruits Th1 cells and cytotoxic T cells to the TME, enhancing anti-tumor immunity.

These secretions of TAMs have dual roles depending on the balance between M1 and M2 phenotypes in the TME. For example, while TNF-α can induce tumor cell death, it can also promote tumor progression by activating NF-κB signaling in tumor cells. IL-6 can promote tumor cell survival and proliferation but can also activate immune cells in certain contexts (13). Additionally, lactate produced by tumor cells and TAMs acts as both a metabolic fuel and a signaling molecule. Lactate lowers the pH in the TME, which supports immune evasion by inhibiting the activity of cytotoxic T-cells and NK cells, while promoting Tregs and MDSCs that foster an immunosuppressive environment. Understanding the functional diversity and plasticity of TAMs is essential for developing therapeutic strategies aimed at reprogramming these cells to enhance anti-tumor immunity.

## 3 Histones and histone modifications in TAMs

Histones, first identified by the German scientist A. Cosell in 1884, are highly alkaline proteins located in the nuclei of eukaryotic cells that package and organize DNA into structural units known as nucleosomes. There are five primary types of histones: H1, H2A, H2B, H3, and H4. Each nucleosome comprises a core octamer consisting of two copies of each of the four core histones, H2A, H2B, H3, and H4, while H1 binds to the DNA between nucleosomes, further compacting the chromatin. Notably, histones H3 and H4 possess long nucleosomal tails that can be covalently modified at various sites (14). Histones play a crucial role in the regulation of gene expression (15). By wrapping DNA around themselves, histones condense the DNA into chromatin, allowing it to fit within the confines of the nucleus. This packaging regulates the accessibility of DNA to transcription factors and other proteins, thereby influencing which genes are activated or repressed (16).

**Figure 2:**
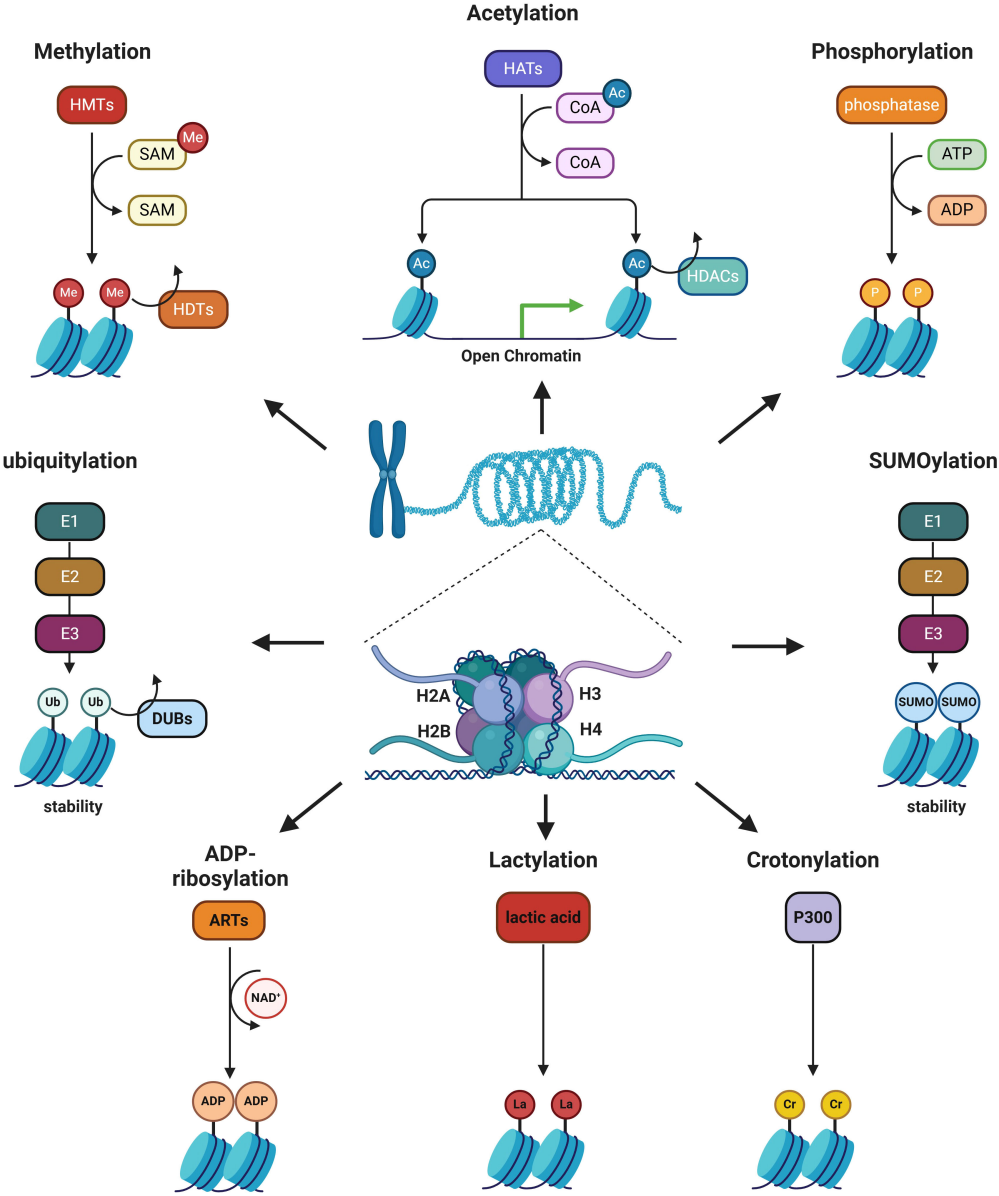
Summary of common histone modifications.

Histone modification is an enzymatic process in epigenetics that involves altering histones through various modifications, including methylation, acetylation, phosphorylation, ubiquitination, SUMOylation, ADP-ribosylation, crotonylation, and lactylation (**Figure 2**). Histone modification can both eliminate and introduce binding sites for specific protein complexes, as well as influence interactions between histones and DNA or other histones, altering the loose or condensed state of chromatin and thereby regulating gene expression (17). Numerous studies have demonstrated that histone modifications play a crucial role in regulating macrophage phenotypes (18, 19). Epigenetic reprogramming of TAMs via targeting histone modifications can shift their polarization from M2 to M1 phenotypes, thereby enhancing anti-tumor immunity and improving the efficacy of cancer immunotherapy.

### 3.1 Histone methylation

Histone methylation refers to the methylation that occurs on the N-terminal arginine or lysine residues of H3 and H4 histones. Arginine can be methylated once (resulting in monomethyl arginine) or twice. Arginine methyltransferases can transfer two methyl groups to the same nitrogen atom of an arginine residue to form asymmetric dimethylarginine, or they can add a methyl group to each N terminus, resulting in symmetrical dimethylarginine (20). Similarly, lysine can be methylated once, twice, or even three times, catalyzed by lysine methyltransferases. Among histone modifications, methylation is the most extensively studied class. Methylation at different sites on histones can produce diverse biological effects. Methylation of H3K4 and H3K36 occurs in genomic regions with transcriptional activity and is involved in the transcriptional activation process (21). H3K9me3/2 and H3K27me3 are commonly found on transposons and inactive genes, where they repress transcription by recruiting protein complexes that bind to nucleosomes and modify chromatin structure. H3K9me3 is enriched in heterochromatin, while H3K9me2 is frequently associated with gene silencing in euchromatin. In summary, the functions of histone methylation are primarily reflected in heterochromatin formation, gene imprinting, X chromosome inactivation, and transcriptional regulation.

#### 3.1.1 Histone methyltransferases and histone methyltransferase complexes

There are two types of histone methyltransferases (HMTs): histone arginine N-methyltransferases and histone lysine N-methyltransferases (22). Irina Tikhanovich et al. reported that protein arginine methyltransferase 1 (PRMT1) as HMT positively regulated peroxisome proliferator-activated receptor γ (*Pparγ*) expression by H4R3me2a at the PPARγ promoter in murine macrophages (23). PPARγ was one of the key transcription factors promoting M2 polarization (24). Moreover, Jie Zhao et al. showed that PRMT1/IL-6/STAT3 axis promoted alcohol-associated hepatocellular carcinoma progression via inducing M2 polarization in mice, and PRMT1 expression was correlated with STAT3 activation in TAMs in human hepatocellular carcinoma specimens (25). Yuyang Du et al. found that ASH1L, an H3K4 methyltransferase, increased the expression of *Ccl2* and *Csf1*, which polarized M2-like pro-tumorigenic macrophages in hepatocellular carcinoma (26). Likewise, Jianchun Wu et al. reported that KMT2D as another H3K4 methyltransferase could increase the expression of *Ccl2*, thereby promoting M2 polarization of TAMs in head and neck squamous carcinoma (27). DOT1L is the only known HMT for H3K79. Xiang Chen et al. found that DOT1L silencing or a DOT1L inhibitor preferentially suppressed the production of IL-6 and IFN-γ but not of TNF-α in macrophages triggered by TLR ligands or virus infection. DOT1L was recruited to the proximal promoter of the *Il6* and *Ifnb1* but not *Tnfα* and then mediated H3K79me2/3 modification at the *Il6* and *Ifnb1* promoters, consequently facilitating the transcription and expression of *Il6* and *Ifnb1* in macrophages (28). From this, Lisa Willemsen et al. found that DOT1L inhibition led to macrophage hyperactivation via down-regulating *Srebf1* and *Srebf2* expression to directly and indirectly control cholesterol and fatty acid synthesis in macrophages (29). These results suggest that HMTs are potential therapeutic targets in regulating M2 polarization of TAMs for cancer immunotherapy (**Table 2**).

**Table 2:** The function of HMTs and their complexes in TAMs.

Gene	Location	HMTs	HMT complexes	Function	Reference
*Pparγ*	H4R3me2a	PRMT1		promoting M2 polarization	(23, 25)
*Ccl2* and *Csf1*	H3K4me3	ASH1L		promoting M2 polarization	(26)
*Ccl2*	H3K4me3	KMT2D		promoting M2 polarization	(27)
*Il6* and *Ifnb1*	H3K79me2/3	DOT1L		production of IL-6 and IFN-γ	(28)
*Srebf1* and *Srebf2*	H3K79me2/3	DOT1L		macrophage hyperactivation	(29)
*Ccl2*	H3K27me3		EZH2	decreased CCL2 expression	(31)
*Hif1α*	H3K27me3		EZH2	promoting M2 polarization	(32)
*Rip1*	H3K4me3		WDR5	promoting M2 polarization	(35)

Besides, a series of HMT complexes also play an important role in regulating macrophage function. The histone methyl transferase enhancer of zeste homolog 2 (EZH2) is a member of HMT and polycomb repressive complex 2, which catalyzes H3K27me3 (30). Several studies have revealed that EZH2 is associated with TAMs. For examples, Yang Zheng et al. found that macrophage infiltration in small-cell lung cancer reduced significantly in a stage-dependent manner, attributed to the decreased expression of CCL2, a potent chemoattractant for monocytes. *Ccl2* expression was inhibited by EZH2-mediated H3K27me3 in the enhancer regions (31). Yan Dong et al. identified that HIF1*α* was activated in TAMs and acted as an important factor for the immune suppressive microenvironment. Meanwhile, epigenetically silencing of *Hif1α* via H3K27me3 in the promoter region was achieved by CRISPR/dCas9-EZH2 system, and the *Hif1α* silenced TAMs manifested as inheritable M1 phenotype in melanoma (32). Furthermore, Cheuk-Him Man et al. found a hitherto undescribed PLK4/PRMT5/EZH2/H3K27me3 axis in TP53-mutated acute myeloid leukemia. The EZH2 mediated H3K27me3 could activate the cGAS-STING pathway, leading to secretion of cytokines and chemokines and activation of macrophages upon coculture with acute myeloid leukemia cells (33). Interestingly, protein methylation of EZH2 also affects TAMs differentiation. WDR5, a vital component of SET/MLL (SET-domain/mixed-lineage leukemia) HMT complexes, played a key role in H3K4me3 and subsequent transactivation of target genes (34). Yan Zhang et al. showed that methionine adenosyltransferase II alpha could up-regulate RIP1 expression by interacting with WDR5 to increase H3K4me3 at promoter regions of *Rip1*, resulting in modulating the activation and maintenance of M2-tpye TAMs in gastric cancer (35). Thus, HMT complexes intervention should be a novel therapeutic strategy in avoiding M2 polarization in TAMs (**Table 2**).

#### 3.1.2 Histone demethyltransferases

In addition to the presence of HMTs, demethylases have been identified. Previously, histone methylation was thought to be stable and irreversible. The discovery of histone demethyltransferases (HDMs), including lysine-specific demethylase, Jumonji domain-containing hydroxylases and peptidyl arginine deiminases, makes histone methylation process more dynamic. Evidences have shown that the expression level of the H3K27 demethylase JMJD3 could be influenced by cytokines and tumor derived exosomes present in the TME, and a high level of JMJD3 contributes to M2 polarization. For example, Jing Xun et al. discovered that breast cancer cells induced TAMs to express more JMJD3 by secreting exosomes containing miR-138-5p, thus enhancing M2 polarization in TAMs (36). However, much research is needed to elucidate the role of HDMs in TAMs.

#### 3.1.3 Others

It has also been reported that protein or long noncoding RNA directly interfere with histone methylation in the form of molecular interactions to regulate the differentiation and function of TAMs. Yang Li et al. identified that NNMT could promote IL-6 and GM-CSF expression by directly decreasing the H3K27me3 levels in *Il6* and *Csf2*, thus promoting differentiation of macrophages into M2-type TAMs and generation of myeloid-derived suppressor cells from peripheral blood mononuclear cells (37). Changhao Chen et al. identified a long noncoding RNA, termed lymph node metastasis associated transcript 1 (LNMAT1), which was upregulated in lymph node-positive bladder cancer. Mechanistically, LNMAT1 epigenetically activated CCL2 expression by recruiting hnRNPL to *Ccl2* promoter, which led to increased H3K4me3 that ensured hnRNPL binding and enhanced transcription. LNMAT1-induced upregulation of CCL2 recruited macrophages into the tumor (38).

In summary, the occurrence of histone methylation which is regulated by the complex HMTs/HDMs enzyme system promotes TAMs M2 polarization. Thus, targeting this enzyme system to regulate specific gene expression could serve as a novel strategy in cancer immunotherapy by expediting TAMs polarization from M2 to M1.

### 3.2 Histone acetylation

Histone acetylation is another crucial post-translational modification that occurs on histone proteins. In this process, an acetyl group is added to the lysine residues of histone proteins, typically at the N-terminal tails (39). This modification is catalyzed by enzymes called histone acetyltransferases (HATs) and removed by histone deacetylases (HDACs). When histones are acetylated, the positive charge of lysine residues is neutralized, reducing the interaction between histones and the negatively charged DNA. This relaxation of chromatin structure creates a more open and accessible configuration, allowing transcription factors and other regulatory proteins to access DNA more easily, thereby promoting gene transcription. Therefore, histone acetylation and deacetylation work in a dynamic balance to regulate chromatin structure and gene expression.

#### 3.2.1 Histone acetyltransferases

So far, about 30 HATs have been identified in humans, which can be divided into two classes: type A and type B, mainly based on their subcellular localization. A-type HATs are located in the nucleus and play a role in histone acetylation of chromatin associated with transcription, whereas B-type HATs are present in the cytoplasm, acetylates newly synthesized histones and affects nucleosome structure. A-type HATs achieved more attraction in histone acetylation and are further divided into five families according to its catalytic domain, including GNAT family, MYST family, CBP/p300 family, TAF1 family, and TIF III C90 family. Functionally, the GNAT family is mainly responsible for the acetylation of Lys sites on histone H3, while the MYST family is related to the acetylation of lysine sites on histone H4, such as H4K16 (40).

HATs can regulate TAMs polarization via promoting the expression of chemokines. For instance, the NOTCH signaling pathway (41) and downstream CCL2/CSF1 (42) are critically involved in TAMs activation and polarization. CREBBP and p300 (encoded by EP300) are two closely related HATs and worked as transcriptional co-activators via H3K27 acetylation, as revealed by germinal center-directed deletion targeting *Crebbp* or EP300 on murine models (43). On this basis, Yaohui Huang et al. reported that the mutation or knockdown of *Crebbp* or EP300 inhibited H3K27 acetylation, downregulated FBXW7 expression, and activated the NOTCH pathway and downstream CCL2/CSF1 expression, resulting in TAMs polarization to M2 phenotype and B-lymphoma cells proliferation both *in vitro* and *in vivo (* 44). Additionally, Yihao Liu et al. showed that the CTCF/PACERR complex could recruit p300, resulting in increased chromatin accessibility and transcriptional activation of PTGS2 that was the critical driver of TAMs M2 polarization in pancreatic ductal adenocarcinoma (45). IL-6, a key interleukin inducing M2 polarization (46), is another potential target regulated by histone acetylation. James A. Rodrigues et al. found that EP300 loss-of-function relieved p300’s transcriptional and/or physical-tethering inhibition on IL-1α signaling, subsequently activating the IL-6/JAK/STAT3 pathway to drive oncogenesis in bladder cancer (47). However, Yichang Wang et al. discovered that ubiquitin-specific peptidase 24 (USP24) increased the level of histone H3 acetylation in the promoter of *Il-6* by stabilizing p300, thereby increasing the IL-6 expression in M2 macrophages to promote the progression of lung cancer (48).

We found that a contradiction arose regarding the role of p300 in IL-6 regulation. On one hand, EP300 loss-of-function relieves its inhibition on IL-1α signaling, activating the IL-6/JAK/STAT3 pathway and driving bladder cancer. On the other hand, USP24 stabilizes p300, increasing histone H3 acetylation at the IL-6 promoter, which enhances IL-6 expression in M2 macrophages and promotes lung cancer progression. Thus, p300 appears to have context-dependent and seemingly opposing roles in IL-6 regulation and TAM polarization. It is valuable to elucidate the regulation of other chemokines by histone acetylation in the future.

#### 3.2.2 Histone deacetylases

Conversely, HDACs remove acetyl groups from histones, leading to chromatin condensation and reduced transcriptional activity. 18 HDACs have been identified in humans and can be divided into two families based on conserved deacetylase domains and their dependence on specific cofactors: the deacetylase family with Zn^2+^ dependence and the sirtuin protein family with NAD^+^ dependence. According to their similarity to yeast deacetylases, the deacetylases family can be subdivided into class I (HDAC1, 2, 3 and 8), class II (HDAC4, 5, 6, 7, 9 and 10) and class IV (HDAC11). Sirtuin proteins are classified as class III HDACs (49). The substrate specificity of HDAC is relatively low. One HDAC can act on multiple substrates, or multiple HDACs can act on the same substrate. They usually bind to each other and interact with other enzymes to act, thereby participating in the regulation of basic cell functions such as cell proliferation, cell cycle, regeneration, apoptosis and differentiation (50). Xiang Zheng et al. showed that suppressing HDAC2 in TAMs resulted in reduced proliferation and migration, increased apoptosis of cancer cell lines and primary lung cancer cells in coculture systems of TAMs and cancer cells. HDAC2 regulated the M2-type TAMs via acetylation of histone H3 and transcription factor SP1. Moreover, myeloid cell-specific deletion of *Hdac2* and pharmacologic inhibition of all class I HDACs in four different murine lung cancer models induced the switch from M2-type to M1-type TAMs (51). In another study, Wenhong Liu et al. found that NEDD9 overexpression inhibited HDAC4 activity to enhance H3K9 acetylation of the *Nedd9* promoter and activation of the FAK/NF-κB signaling pathway, leading to promote IL-6 secretion, which further drives breast cancer progression. Moreover, NEDD9 activation fostered the M2 macrophage polarization in the TME (52). Interestingly, Haruka Shinohara et al. reported that colorectal cancer-derived extracellular vesicles containing miR-145 polarized macrophage-like cells into the M2-like phenotype through the downregulation of HDAC11. These extracellular vesicles treated macrophages caused significant enlargement of the tumor volumes (53). HDACs have inconsistent roles in regulating TAMs polarization. From the past studies, HDAC2 itself had a positive effect on M2 polarization, whereas HDAC4 and HDAC11 had the negative effects on M2 polarization, suggesting that increasing HDAC4 and HDAC11 expression and decreasing HDAC2 expression are targets for tumor immunotherapy.

Collectively, HATs such as CREBBP and p300, promote M2 polarization by activating pathways like NOTCH/CCL2/CSF1 and IL-6/JAK/STAT3, though p300 exhibits context-dependent roles in IL-6 regulation. Conversely, HDACs remove acetyl groups, condensing chromatin and reducing transcription, with HDAC2 promoting M2 polarization while HDAC4 and HDAC11 inhibit it. These opposing effects highlight the complex, context-specific roles of HATs and HDACs in TAM polarization, suggesting that targeting specific enzymes could offer therapeutic potential for tumor immunotherapy. Further research is needed to clarify these mechanisms.

### 3.3 Histone lactylation

The progress of lactyl groups adding to histone proteins is called histone lactylation. This modification was first discovered in 2019 and is closely associated with cellular metabolic states, particularly those related to lactate production (54). Lactate metabolism in tumor cells is a key feature of TME, often driven by the “Warburg effect”, where tumor cells preferentially utilize glycolysis over oxidative phosphorylation, even in the presence of oxygen (55). This metabolic reprogramming leads to high production of lactate, which accumulates in the TME and influences both tumor growth and the behavior of surrounding stromal and immune cells (56). Lactate can lead to histone lactylation within TAMs, directly modifying gene expression to support tissue repair and immunosuppressive pathways. In the TME, lactate metabolism by tumor cells promotes TAMs polarization toward a M2-like phenotype, which is partly due to lactate-induced signaling pathways, such as stabilization of hypoxia-inducible factor-1α (HIF-1α) and activation of specific metabolic pathways within TAMs that favor an immunosuppressive state. Jialiang Cai et al. found that lactate produced by tumors induced lactylation of the histone H3K18la site upon transport into macrophages, thereby activating transcription and enhancing M2-like TAMs activity (57). Xia Fang et al. put forward that HIF-1α stabilization and histone lactylation were both required for M2-like TAMs polarization (58). Besides, Xin Wu et al. showed that the secretion of lactate and histone lactylation alterations within tumor cells recruits and polarizes M2-like TAMs through the PI3K/AKT-CXCL14 axis in neuroblastoma (59).

Lactate enhances the immunosuppressive properties of TAMs by promoting the expression of anti-inflammatory cytokines like IL-10 and TGF-β. Alessandra De Leo et al. found inhibition of glycolysis or lactate production in monocyte-derived macrophages (MDMs) impaired IL-10 expression and T cell suppression. Mechanistically, intracellular lactate-driven histone lactylation promoted IL-10 expression to suppress T cell activity. Consistently, abrogated histone lactylation led to the accumulation of intratumoral T cells and tumor growth delay. Besides, inhibition of histone lactylation combination with immunotherapy could block glioblastoma (GBM) progression *in vivo (* 60). Xiuming Li et al. reported that tumor-derived lactate fuels H3K18 lactylation prohibited RARγ transcription in macrophages, consequently enhancing IL-6 levels in the TME and endowing macrophages with tumor-promoting functions via activation of signal transducer and activator of transcription 3 (STAT3) signaling in colorectal cancer cells (61).

Given these multifaceted roles, histone lactylation in the TME is considered a promising target for cancer therapies aimed at disrupting the metabolic adaptations of tumors and reshaping the immune landscape to improve anti-tumor immune responses. Kiranj Chaudagar et al. demonstrated that combining androgen deprivation therapy (ADT), PI3K inhibitors (PI3Ki), and PD-1 antibodies (aPD-1) suppressed H3K18 lactylation and fully activated TAMs, achieving significant therapeutic effects in prostate cancer (62) (63).

### 3.4 Histone phosphorylation

Phosphorylation usually occurs on serine, threonine and tyrosine residues, and is a reversible modification regulated by phosphorylase or kinase, in which the enzyme transfers the phosphate group on ATP to or removes it from the receptor amino acid residue (64). In addition, phosphorylation modification of arginine and histidine is also present, but the biochemistry is less clear because of their instability. Most histone phosphorylation occurs in the N-terminal tail and mainly in histone H3, but the phosphorylation of histone H1 at multiple sites has been demonstrated as one of the prerequisite steps for gene induction (65). Histone phosphorylation may affect chromatin structure and function through two mechanisms. On the one hand, phosphorylation provides a negative charge for histones, which weakens their ability to bind to DNA and loosens chromatin, similar to acetylation modification. On the other hand, protein complexes that specifically recognize phosphorylation sites can recognize and bind to the surface of phosphorylated histones to exert regulatory functions together (66).

Histone phosphorylation regulates the function of TAMs mainly through the intervention on the interleukin promoter. Sayantan Banerjee et al. reported that both the lack of transcription favorable histone phosphorylation at the *Il-12* promoter and the abundance of ERK1/2-dependent histone phosphorylation at the *Il-10* promoter led to the polarization of TAMs to M2, although the mechanism underlying the ability of the TME to preferentially change the phosphorylation pattern of histones in TAMs has not been clarified (67). Interestingly, Oakley C Olson et al. reported that TAMs suppressed the duration of Taxol-induced mitotic arrest in breast cancer cells and promote earlier mitotic slippage. This correlates with a decrease in the phosphorylated form of H2AX, decreased p53 activation, and reduced cancer cell death in interphase (68).

### 3.5 Histone ubiquitination

Protein ubiquitination is a 76-amino acid ubiquitin molecule (Ub) connected to the Lys of the target protein through the C-terminus. Target proteins can undergo either mono-or polyubiquitination, which is mediated by a combination of E1 activating enzymes, E2 conjugating enzymes, and E3 ligases, while deubiquitinating enzymes (DUBs) can remove Ub from the target protein (69). Histones are among the most abundant mono-ubiquitinated proteins (70). Wenlai Zhou et al. found that mono-ubiquitination of H2A at Lys 119 prevented the recruitment of SPT16 and SSRP1 at the transcriptional promoter region, and blocked RNA polymerase II release at the early stage of elongation, which mediated selective repression of a specific set of chemokine genes modulating migratory responses to TLR activation in macrophages, such as *Ccl5*, *Cxcl0*, and *Cxcl12 (* 71).

Histone ubiquitination can promote its degradation, participate in DNA damage repair and chromatin remodeling, thereby regulating gene transcriptional activation and silencing. The regulatory effect of histone ubiquitination on TAMs is reflected in its stability to HMTs and HATs. For examples, Peng Wang et al. identified SET and MYND domain-containing protein 3 (SMYD3) as the methyltransferase of EZH2 at K421 residue which accelerates EZH2 ubiquitin proteasome degradation to promote M2 polarization in gastric cancer (72). Lingfang Du et al. revealed that epigenetic regulator KDM6B by virtue of its demethylase activity prevented M2 polarization via promoting the intranuclear degradation of β-catenin (73). Yichang Wang et al. found that USP24 could stabilize p300 to increase the levels of histone-3 acetylation and NF-κB, thereby increasing IL-6 transcription in M2-type TAMs and lung cancer cells, resulting in cancer malignancy finally (48).

### 3.6 Histone SUMOylation

SUMOylation is another reversible post-translational modification of proteins, which is also known as small ubiquitin-like modification protein. Similar to ubiquitination, SUMO is a small protein containing 100 amino acids. It is transferred to lysine residues of the target protein by SUMO E1, E2 and E3 enzyme, while SUMO specific protease (SENP) can remove SUMO modification from the target protein (74). Surprisingly, Qi Yang et al. found that HDAC4, belonging to the SUMO E3 ligase family, negatively regulated NF-κB activation for IκBα SUMOylation (75).

Currently, five SUMO isoforms have been identified in humans, namely SUMO-1, SUMO-2, SUMO-3, SUMO-4 and SUMO-5. SUMOylation is functionally different from ubiquitination (76). Ubiquitination mainly promotes the degradation of the target protein, while SUMO modification makes the protein more stable. SUMO modification is essential for biological processes such as gene expression regulation, DNA damage repair, maintenance of genome integrity, cell cycle and apoptosis. Histone SUMOylation was discovered in 2003 (77), but there is still relatively little research and understanding on this aspect. It is known that SUMO can conjugate all histone proteins, H2A variant H2A.X and H3 variant Cse4, and some modification sites have been identified, such as H2BK6, K7, H4K5, K8, etc (78). Histone SUMOylation, which is commonly associated with transcriptional repression, can lead to chromatin condensation and gene silencing by promoting the recruitment of the deacetylase HDAC1 and the heterochromatin-related protein HP1 (79, 80). In addition, acetylated H4 can induce SUMO-1 binding, and enhance SUMOylation through the interaction of acetyltransferase P300, indicating that acetylation may promote SUMOylation (81), and there is a mutual regulatory relationship between SUMOylation and different histone modifications. Gabriel Pascual et al. reported that ligand-dependent SUMOylation of the PPAR-γ ligand-binding domain could target HDAC3 complexes on inflammatory gene promoters in mouse macrophages, thereby antagonizing inflammatory (82).

### 3.7 Histone ADP-ribosylation

ADP-ribosylation uses NAD^+^ as a substrate and adp-ribosyltransferase (ARTs) catalyzes the transfer of ADP-ribose groups to the side chain of amino acid residues of target proteins, which regulates the structure and function of proteins transfers. ADP-ribosylation is modified in two forms: mono-ADP-ribosylation and poly-ADP-ribosylation, catalyzed by ecto-ARTs and poly-ARTs.

ADP-ribosylation of all core histones and linker H1 can occur on a variety of amino acid residues such as glutamate, aspartic acid, arginine and lysine in the histone tail, of which 1% occurs on the lysine residue (83). Studies have shown that histone ADP-ribosylation is involved in the regulation of higher chromatin structure and can promote chromatin relaxation (84). Histone ADP-ribosylation directly destabilizes histone-DNA interactions in the nucleosome and increases the site accessibility of the nucleosomal DNA to nucleases (85). Histone H2B and H3 were mainly involved in DNA damage repair, and a small part of H1 and H4 were also involved. DNA damage increases PAR modification of histones by a mechanism that remains unclear. In addition, the level of ADP-ribosylation of histones increases during cell proliferation and continues throughout the proliferation process, contributing to DNA assembly. C Wang et al. found that M-CSF induced ADP-ribosyltransferase diphtheria toxin-like 1 (ARTD1) poly-ADP-ribosylation in macrophages (86). Ricardo Martinez-Zamudio et al. reported that LPS stimulation-induced ADP-ribosylation at the nucleosome-occupied promoters of *Il-1β*, *Mip-2*, and *Csf2* could facilitate the transcription of these genes in macrophages (85). It can be seen that M1-type TAMs are activated in a manner that generally raises the level of histone ADP-ribosylation.

### 3.8 Histone crotonylation

Histone crotonylation is a post-translational modification where a crotonyl group (derived from crotonic acid) is added to lysine residues on histone proteins (87). Discovered in 2011, this modification is structurally distinct from acetylation (88), but Benjamin R Sabari et al. showed that the coactivator p300 has both crotonyltransferase and acetyltransferase activities, and that p300-catalyzed histone crotonylation directly stimulated transcription to a greater degree than histone acetylation (89). Histone crotonylation is associated with active gene promoters and enhancers. Recently, histone crotonylation is investigated in relation to cancer and immune regulation, where it appears to contribute to cellular plasticity and adaptation in the macrophage. For examples, Jing Yang et al. firstly showed the existence of crotonylation in porcine alveolar macrophages (90). Yu Zou et al. reported that inhibition of p300 alleviated partial infraorbital nerve transection-induced macrophage activation and reduced the expression of inflammatory cytokines *Tnfα* and *Il1β*, as well as chemokines *Ccl2* and *Cxcl10*. Correspondingly, exogenous crotonyl-CoA induced macrophage activation and the expression of *Tnfα, Il1β, Il6, Ccl2* and *Ccl7* in trigeminal ganglia (91). Similarly, Lingzhi Li et al. found that a crotonyl-CoA-producing enzyme ACSS2 (acyl-CoA synthetase short chain family member 2) remarkably increased H3K9 crotonylation (H3K9cr) level without influencing H3K9ac in kidneys and tubular epithelial cells. Furthermore, genetic and pharmacologic inhibition of ACSS2 both suppressed H3K9cr-mediated IL-1β expression, which thereby alleviated IL-1β-dependent macrophage activation and tubular cell senescence to delay renal fibrosis (92). Christopher McCrory et al. reported that the short-chain fatty acid crotonate increases histone crotonylation, reduces hyphal formation within macrophages, and slows macrophage lysis and immune escape of *C. albicans (* 93). Hao Zhang et al. showed a proteome-wide crotonylation profile of human leukemia monocyte cell line (THP1 cells) infected with methicillin-resistant *Staphylococcus aureus* and further treated with vancomycin, which pointed to a comprehensive understanding of the biological functions of histone crotonylation in human macrophages (94). Given its distinct and functionally relevant effects, histone crotonylation is a promising focus for epigenetic research and potential therapeutic strategies. Based on the fact that inhibition of histone crotonylation reduces cytokines and chemokines secreted by M2b-type TAMs, we hypothesized that crotonylation promotes M2 polarization of TAMs.

### 3.9 Other histone modification

Histone modifications play a key role in physiological and pathological regulation, which is of great value for basic research and clinical exploration. Of course, there are more propionylation, butolylation, hydroxylation and formylation, etc. Interested partners can go to the relevant information to understand. In summary, there are many kinds of histone modifications, and different types have different degrees of crosstalk. Although the role of the aforementioned histone modifications in the function of TAMs has not been thoroughly investigated, based on their impacts on macrophages in other diseases, we believe that they will become potential targets in tumor immunotherapy via regulating TAMs polarization.

## 4 Crosstalk between different histone modifications

Crosstalk between different histone modifications plays a crucial role in the regulation of gene expression and chromatin dynamics. Yifat Geffen et al. used the most advanced mass spectrometry to analyze the largest collection of proteogenomics data from 1,110 patients across 11 cancer types, and found that acetylation and phosphorylation often occurred in close proximity and could influence each other’s functions. For instance, phosphorylation at serine 31 may stimulate acetylation through the activity of the p300 acetyltransferase. Similarly, S28 phosphorylation may reduce trimethylation at K27, thereby priming the site for acetylation (95). Anja Armache et al. reported that phosphorylation of the histone variant H3.3 at serine 31 occurred in response to stimulation, facilitating rapid gene transcription by recruiting the histone methyltransferase SETD2 and ejecting the corepressor ZMYND11, thereby enabling preferential access to the transcription machinery (96). Besides, Simone Tamburri et al. found that the loss of H2AK119ub could induced a loss of H3K27me3 deposition (97), and Schulze JM et al. found that H2BK123ub promoted the methylation of histone H3 at lysine 4, 46, and 79 (98). Cathy J Spangler et al. found that ubiquitylation of histone H2B lysine 120 (H2BK120ub) could stimulate DOT1L methylates histone H3 lysine 79 during transcriptional elongation in a classical trans-histone crosstalk pathway (99).

As mentioned earlier in 2.8, p300 also participates in histone crotonylation, however histone crotonylation activity is much less efficient compared to histone acetylation due to steric constraint (100). Also, loss of HDAC1/2 led to enrichment of H3K18cr around transcription start sites, which largely overlapped with H3K18ac and correlated with gene activity (101). HDAC4 belongs to the SUMO E3 ligase family, and Qi Yang et al. revealed that HDAC4 could directly sumoylate IκBα to inhibit NF-κB activation (75). Histone acetylation and lactation may compete for the same lysine site and produce different regulatory effects (102). Interestingly, Rongxuan Zhu et al. have recently discovered that acetyl-CoA synthetase 2 functions as a bona fide lactyl-CoA synthetase and converts lactate to lactyl-CoA, further mediating histone lactation (103).

It can be seen that histone methylation and acetylation are the basis for regulating gene expression. When histone modification crosstalk occurs, other modifications still mainly act by affecting methylation and acetylation. Of course, at present, the researches on the histone modification crosstalk are still not mature, the main reason is the lack of research technology. In the future, it may be a good direction to apply PTM-CrossTalkMapper to study histone modification crosstalk (104).

## 5 Metabolic reprogramming and histone modifications in the tumor-associated macrophages

Nowadays, the metabolic reprogramming of TAMs is a research hotspot (105). M1-tpye TAMs mainly rely on glycolysis for energy supply, and M2-tpye TAMs mainly rely on OXPHOS from fatty acid oxidation to supply energy (106). Metabolic changes in TAMs can directly influence histone modifications by altering the availability of metabolites that serve as cofactors or substrates for epigenetic enzymes. For example, Manjula Karpurapu et al. reported that activation-induced cytidine deaminase-mediated active demethylation of the *Klf4* promoter was essential for PU.1-dependent transcriptional regulation of *Klf4*, enabling monocyte/macrophage differentiation, as promoter methylation inhibited PU.1 binding and *Klf4* expression (107, 108). Furthermore, α-Ketoglutarate and Succinat regulate the activity of histone demethylases (e.g., Jumonji domain-containing proteins) and DNA hydroxylases. S-adenosylmethionine as the primary methyl donor for HMTs, of which levels are influenced by methionine and folate metabolism. Altered levels of α-Ketoglutarate and succinate or changes in S-adenosylmethionine availability can both affect histone methylation and gene expression in TAMs. The production and secretion of pro-inflammatory cytokines (IL-6, IL-1, and TNF-α) through macrophages are also controlled by the obtainability of glutamine. The accumulation of PGE2 secreted by tumor cells can transform TAMs M1 to M2 (109).

More importantly, the “Warburg effect” of tumor means that tumor cells consume a lot of glucose and produce large amount of lactic acid even in the presence of sufficient oxygen. Lactic acid accumulates in cells and then is exported into the extracellular environment via activating monocarboxylate transporters (MCTs) on cell membrane, which ultimately results in establishing an acidic TME and regulating histone lactation in the TAMs. Xiaowei She et al. discovered that SETDB1-mediated tri-methylation of MCT1 at K473 stabilized MCT1 by blocking Tollip-mediated autophagic degradation, promoting tumor glycolysis, M2-like TAMs polarization, and lactate shuttle activity in colorectal cancer (110). Na Liu et al. also found that lactate produced by glycolysis of tumor cells in the tumor microenvironment activates mTOR pathway, thereby phosphorylating the transcription factor TFEB and inhibiting its nuclear translocation, thereby inhibiting the expression of ATP6V0d2 in TAMs. The inhibition of ATP6V0d2 could mediate HIF-2α lysosomal degradation and program TAMs to M2 polarization (111). Currently, it is generally believed that the increase in lactate and histone lactation levels promotes TAMs M2 polarization.

Additionally, the cells in the TME constantly compete for nutrients such as glucose and oxygen, and redirect cellular metabolism from oxidative respiration to anaerobic glycolysis, which reduces the production of acetyl-coenzyme A, further influencing histone acetylation (112). Mario A. Lauterbach et al. discovered that macrophages increased glycolysis and tricarboxylic acid cycle volume to generate more acetyl-coenzyme A from glucose upon TLR4 activation, thus leading to augmented histone acetylation which facilitated the transcription of LPS-inducible gene sets contributing to M1 polarization (113). Zhen Dong et al. reported that glycolytic metabolism enhanced histone acetylation, particularly H3K9 acetylation, to promote IL-1β production in M1-type TAMs (114).

**Figure 3:**
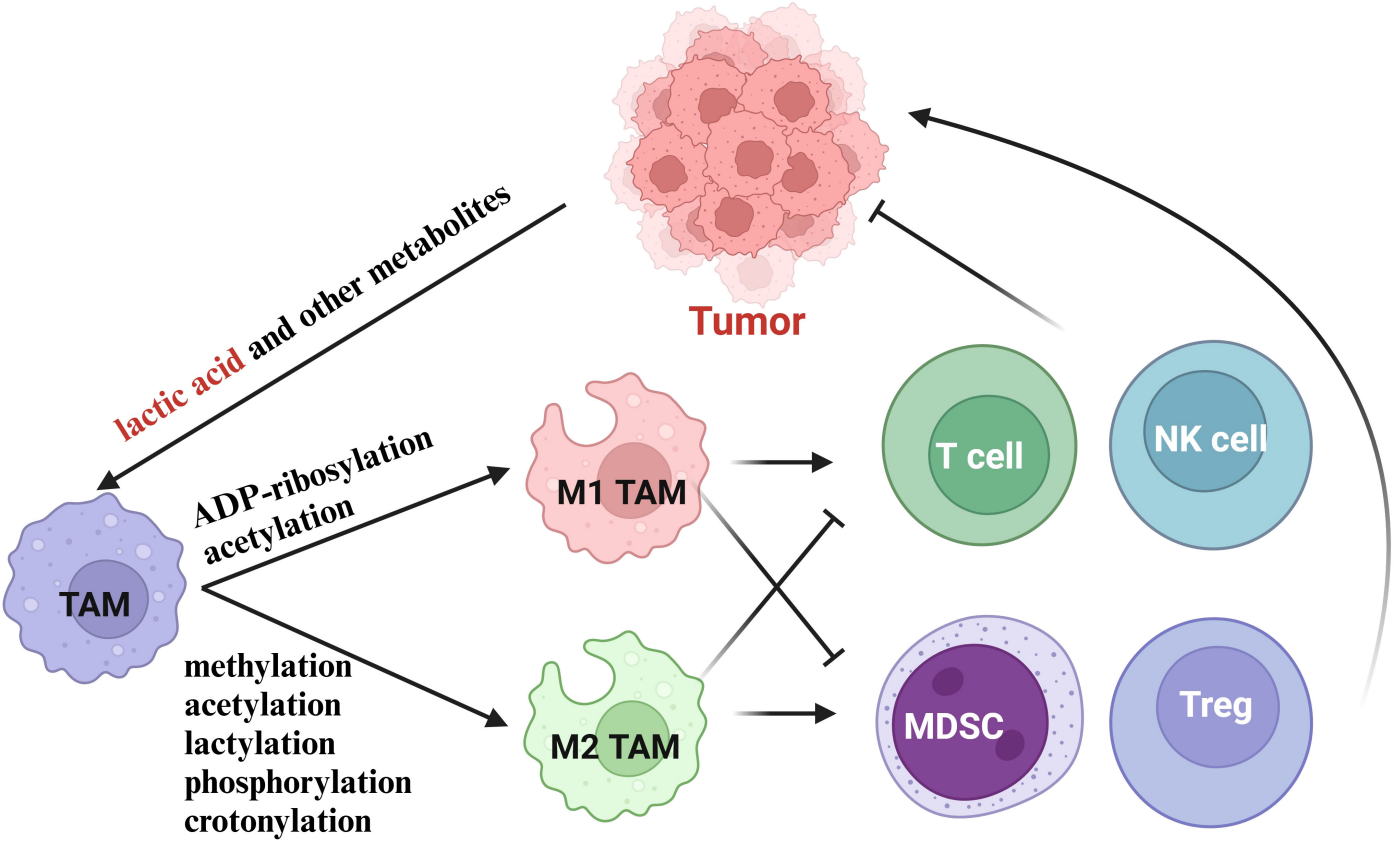
The effects of histone modifications in TAMs for cancer immunotherapy.

In summary, the metabolites of various cells in TME can affect the polarization level of TAMs. Among them, histone lactylation, driven by lactate accumulation in the TME, promotes M2 polarization, while histone acetylation, influenced by metabolic shifts and acetyl-coenzyme A availability, supports M1 polarization. These findings highlight the intricate interplay between metabolism, epigenetics, and immune cell function in the tumor microenvironment (**Figure 3**).

## 6 Histone modifications and cancer immunotherapy

The level of histone modification can directly serve as a screening indicator for tumors to some extent. For instance, Lei Fan et al. constructed a prognosis-related histone phosphorylation regulated genes signature, and found that histone phosphorylation regulated genes risk score was closely related to the prognosis of hepatocellular carcinoma, tumor immune process and tumor cell progression (115).

HDAC is also an important factor in tumor progression. Zhi Yang et al. revealed that the expression of HDAC3 was upregulated in clinical gastric cancer tissues. Moreover, HDAC3 promoted gastric cancer progression by degrading FOXA2, which in turn activates the FTO/m6A/MYC signaling axis *in vitro* and *in vivo (* 116). Thus, a promising strategy for cancer treatment is normalizing abnormal epigenetic signatures through agents like histone deacetylase inhibitors (HDACis). HDACis have shown significant potential in cancer immunotherapy by enhancing the immune system’s ability to recognize and attack tumor cells. They promote T-cell infiltration into tumors and improve the efficacy of immune checkpoint inhibitors (ICIs), such as PD-1/PD-L1 antibodies (117, 118). HDACis can also help overcome resistance to ICIs by modulating the TME. They reduce the immunosuppressive effects of Tregs and MDSCs, thereby enhancing anti-tumor immune responses. Combining HDACis with ICIs has shown improved clinical outcomes in a serious of cancers, including colorectal cancer (119)and non-small cell lung cancer (120). However, HDACis has off target effects that greatly reduce the efficacy. To reduce off-target effects and toxicity, selective HDAC inhibitors targeting specific HDAC isoforms (e.g., HDAC8 (121)) have been developed. These selective inhibitors offer improved therapeutic windows and reduced side effects compared to pan-HDAC inhibitors. Furthermore, HDAC-based dual-target inhibitors, which simultaneously inhibit HDACs and other cancer-related pathways (e.g., PD-L1, SHP2, HSP90), have shown enhanced anti-tumor efficacy. These inhibitors address multiple pathways involved in tumor growth and immune evasion, providing a more comprehensive treatment approach. Interestingly, proteolysis-targeting chimeras was developed to selectively target and degrade specific HDAC isoforms, overcoming limitations of traditional HDAC inhibitors, such as drug resistance and off-target effects (122). More importantly, several HDAC inhibitors, such as vorinostat and romidepsin, have been approved for clinical use in treating cancers like cutaneous T-cell lymphoma (123). Ongoing clinical trials are exploring the use of HDACis in combination with other therapies to improve response rates and overcome resistance in various cancers.

In addition to HDACis, histone methyltransferase inhibitors (HMTis) are also able to combine with ICIs in cancer immunotherapy. For example, Jiaqi Huang et al. reported that the EZH2 inhibitor (EPZ‐6438) enhanced PD-L1 expression and protein stability via upregulating USP22 expression. Importantly, a combination of EPZ‐6438 with anti-PD-1 immune checkpoint blockade therapy improves the TME, enhanced sensitivity to immunotherapy, and exerted synergistic anticancer effects (124). Besides, Yibin Yang et al. reported that increased DOT1L triggered epithelial-mesenchymal transition-mediated metastasis from data regarding 410 patients with human hepatocellular carcinoma, but its targeting *in vivo* was hindered by TAMs-mediated NF-κB signaling, suggesting combined therapy with TAMs depletion or NF-κB inhibition enhanced the efficacy of DOT1L-targeted epigenetic reprogramming (125).

According to the metabolic reprogramming in the TAMs, some metabolic enzymes can also serve as targets for cancer immunotherapy in combination with ICIs. For instance, Tommaso Scolaro et al. reported that upregulation of cytidine deaminase in immunotherapy-resistant tumors increases extracellular uridine diphosphate, which hijacks immunosuppressive TAMs via P2Y6, and targeting cytidine deaminase or P2Y6 disrupts TAM-mediated immunosuppression, enhancing T cell infiltration and restoring anti-PD-1 efficacy in resistant cancers (126). Additionally, epigenetic remodeling holds potential to overcome challenges in CAR T-cell therapy, such as T-cell exhaustion and infiltration barriers. Advances in epigenome editing, utilizing tools like dCas9 and zinc finger proteins, aim to precisely target and regulate gene expression, offering a pathway for site-specific epigenetic therapies.

## 7 Future perspective and challenges

Targeting histone modifications and TAMs holds significant promise for advancing cancer immunotherapy. By reprogramming TAMs from immunosuppressive M2 phenotypes to anti-tumor M1 states, we can enhance the immune system’s ability to combat tumors. Precision epigenetic therapies, such as selective HDACis and HMTis, offer the potential to modulate TAM function with minimal off-target effects. Combining these epigenetic modulators with ICIs or adoptive cell therapies like CAR T-cells could overcome resistance and improve clinical outcomes. Additionally, targeting metabolic pathways in TAMs, such as glycolysis or lactate production, may disrupt their immunosuppressive functions and promote M1 polarization.

However, several challenges must be addressed to realize the full potential of these approaches. The complexity of histone modification crosstalk and the dynamic plasticity of TAMs in the TME complicate therapeutic targeting. Tumor heterogeneity and the diversity of TAM subsets further necessitate tailored strategies for different cancer types. Current studies predominantly employ *in vitro* systems or simplistic murine models, which may not fully capture the complexity of the human TME. There is a critical need for more sophisticated animal models that more accurately mimic human tumors, incorporating aspects such as heterogeneity, extracellular matrix components, and immune cell interactions. Models that enable real-time observation of histone modifications and their effects on TAM function in dynamic environments could yield transformative insights and guide therapeutic strategies. Off-target effects and toxicity associated with broad-spectrum epigenetic inhibitors remain significant hurdles, underscoring the need for isoform-specific inhibitors or targeted delivery systems. Resistance mechanisms, such as compensatory upregulation of alternative pathways, also pose challenges, highlighting the importance of combination therapies. Finally, translating preclinical findings into clinical practice requires large-scale trials to validate the safety and efficacy of these novel approaches.

Given the multifaceted nature of histone modifications and their impact on TAMs, fostering interdisciplinary collaborations among oncologists, immunologists, biochemists, and molecular biologists is essential. Such partnerships can catalyze innovative approaches to decipher the complex interactions within the TME. For instance, combining techniques from systems biology, computational modeling, and high-throughput screening can facilitate the discovery of novel regulators of histone modifications in TAMs.

Despite these challenges, the integration of epigenetic, metabolic, and immunotherapeutic strategies offers a promising path forward. Advances in epigenome-editing technologies, biomarker development, and multi-omics analyses will be critical for identifying optimal therapeutic targets and personalizing treatments. By addressing these challenges, we can harness the power of histone modifications and TAM reprogramming to reshape the TME, enhance anti-tumor immunity, and improve outcomes for cancer patients.
